# Commentary: A Systematic Evaluation of Single Cell RNA-Seq Analysis Pipelines

**DOI:** 10.3389/fgene.2020.00941

**Published:** 2020-09-04

**Authors:** Koji Kadota, Kentaro Shimizu

**Affiliations:** ^1^Graduate School of Agricultural and Life Sciences, The University of Tokyo, Tokyo, Japan; ^2^Collaborative Research Institute for Innovative Microbiology, The University of Tokyo, Tokyo, Japan; ^3^Interfaculty Initiative in Information Studies, The University of Tokyo, Tokyo, Japan

**Keywords:** differential expression analysis, normalization, bulk RNA-seq, scRNA-seq, asymmetry/asymmetric, transcriptome, gene expression

RNA sequencing (RNA-seq) is a common tool for obtaining data related to gene expression (Mortazavi et al., 2008). Identification of genes exhibiting differential expression (DE) in different groups or conditions is critical to analysis of RNA-seq data (Osabe et al., 2019). Recently, Vieth et al. (2019) evaluated a total of 3,000 possible single-cell RNA-seq (scRNA-seq) analysis pipelines, encompassing the entire analytical process—from library preparation protocols to identification of DE genes. By performing a simulated analysis to compare two-group data under various conditions, they found that method of normalization and choice of library preparation protocol had the greatest impact on the outcome of scRNA-seq analyses. Though we agree with the main conclusion, the stated motivation for the research is insufficient and misleading to readers. In short, Vieth et al. neglect the contributions of previous studies based on bulk RNA-seq. In this commentary, we provide facts about what they claim as the differences between scRNA-seq and bulk RNA-seq when performing DE analysis.

There are two main criticisms. First, Vieth et al. state, “One main assumption in traditional DE-analysis is that differences in expression are symmetric.” They subsequently state, “This implies that either a small fraction of genes is DE while the expression of the majority of genes remains constant or similar numbers of genes are up- and down-regulated so that the mean total mRNA content does differ between groups.” Finally, they state, “This assumption is no longer true when diverse cell types are considered.” The second half of the second sentence is probably wrong. Unless they write “the mean total mRNA content does NOT differ between groups,” the relationship with the surrounding text is not logical. Importantly, the asymmetry is already addressed by some previous studies with bulk RNA-seq (Kadota et al., 2012; Evans et al., 2018). Second, as an example, Vieth et al. mentioned an scRNA-seq study that found up to 60% DE genes and differing amounts of total mRNA levels between cell types (Zeisel et al., 2015) for distinguishing scRNA-seq from bulk RNA-seq. However, even the tendency to obtain a large number of DE genes between cell types cannot distinguish these. For example, a bulk RNA-seq dataset exists (Schurch et al., 2016) that can produce nearly 70% DE genes (Zhao et al., 2018). A common feature of these data sets is a high number of replicates (>40 replicates per group). A typical number of cells per cell type in scRNA-seq corresponds to a very large number of replicates per group in bulk RNA-seq. Therefore, a necessary condition for obtaining many DE genes would be the number of replicates.

Regarding the first criticism, we previously showed the need for asymmetry and developed a robust normalization method (dubbed TbT) for manipulation in both symmetric and asymmetric scenarios (Kadota et al., 2012). Although TCC, the R package (Sun et al., 2013) that implements both TbT and DEGES (the generalized form of TbT), evaluated a limited extent of scenarios (~25% DE), Evans et al. (2018) covered the shortfall in an analysis of approximately 5–95% DE when both symmetric and asymmetric scenarios were evaluated. Although Evans et al. (2018) did not perform many replicates in their simulation settings (~five replicates in Figures 7, 8), they still provide important suggestions for asymmetry conditions. Notably, DEGES outperforms the other methods at ~60% DE conditions; this is included in the simulation scenarios of Vieth et al. (2019). Despite citing the paper of Evans et al., Vieth et al. (2019) added only the *representative* bulk methods, TMM (Robinson and Oshlack, 2010) and MR (Anders and Huber, 2010), in their comparison and recommended the use of scran (Lun et al., 2016), which has been developed specifically for scRNA-seq. This is misleading to the reader because *representative* methods are not always accurate. Researchers should thoroughly investigate the most accurate method for given simulation conditions for inclusion in comparative analyses, and make conclusions/recommendations based on the outcomes. We expect the recommendations from Vieth et al. would be different if they had honestly compared the *best* bulk method (i.e., DEGES) as well as the *representative* bulk methods (i.e., TMM and MR).

Related to the second criticism, Vieth et al. (2019) found that relatively straightforward DE-testing methods adapted from bulk RNA-seq perform well with scRNA-seq data and reasoned that scRNA-seq data obtained from unique molecular identifier (UMI) counting are well fit to a negative binomial (NB) distribution (Vieth et al., 2017, 2019). Along with other recent reports (e.g., Van den Berge et al., 2018), it is becoming more apparent that there is no need to distinguish between scRNA-seq and bulk RNA-seq data, at least in DE analysis. Still, some researchers may believe that the high frequency of zero values (i.e., zero-inflation) in scRNA-seq data obtained from tools like Smart-seq2 (Picelli et al., 2014) is a main characteristic that distinguishes bulk RNA-seq data. Nevertheless, many researchers are probably not aware that characteristic zero-inflation has already been found in bulk RNA-seq data with large number of replicates (Esnaola et al., 2013). To the best of our knowledge, the report by Esnaola et al. is the first one describing the need to consider zero-inflation; the authors employed the Poisson-Tweedie family of distributions to consider both zero-inflation and heavy tail behavior. In our opinion, the contributions of Esnaola et al. should be cited when discussing zero-inflation (e.g., Tang et al., 2015).

Taken together, there is no special reason to distinguish between scRNA-seq and bulk RNA-seq, especially in DE analysis. Despite the advances in experimental technology from bulk RNA-seq to scRNA-seq, universally applicable algorithms do exist.

## Author Contributions

KK drafted the manuscript. KS supervised the critical discussion and refined the paper. All authors read and approved the final manuscript.

## Conflict of Interest

The authors declare that the research was conducted in the absence of any commercial or financial relationships that could be construed as a potential conflict of interest.
